# Cerebral Aneurysm Characteristics and Surgical Outcomes: An In-Depth Analysis of 346 Cases Operated Using Microsurgical Clipping

**DOI:** 10.7759/cureus.56933

**Published:** 2024-03-26

**Authors:** Corneliu Toader, Cristian Kakucs, Nicolaie Dobrin, Razvan-Adrian Covache-Busuioc, Bogdan-Gabriel Bratu, Andrei Adrian Popa, Luca-Andrei Glavan, Antonio-Daniel Corlatescu, Matei George Nicolae Grama, Horia-Petre Costin, Alexandru Vladimir Ciurea

**Affiliations:** 1 Department of Neurosurgery, “Carol Davila” University of Medicine and Pharmacy, Bucharest, ROU; 2 Department of Neurosurgery, National Institute of Neurology and Neurovascular Diseases, Bucharest, ROU; 3 Department of Neurosurgery, “Transilvania” University of Brasov, Faculty of General Medicine, Brasov, ROU; 4 Department of Neurosurgery, Clinical Emergency Hospital of Brasov, Brasov, ROU; 5 Department of Neurosurgery, Clinical Emergency Hospital “Prof. Dr. Nicolae Oblu”, Iasi, ROU; 6 Department of Research and Development, Syndical.io, Bucharest, ROU; 7 Department of Neurosurgery, Sanador Clinical Hospital, Bucharest, ROU

**Keywords:** hemodynamic strain, glasgow out-come scale, vasospasm, computed tomography angiography, magnetic resonance angiography, risk factors, epidemiology, surgical treatment, subarachnoid hemorrhage, intracranial aneurysms

## Abstract

This comprehensive study analyzes 346 surgically treated intracranial aneurysms, emphasizing the importance of understanding risk factors and prevalent characteristics in patients. Intracranial aneurysms, primarily of the saccular or berry type, significantly contribute to nontraumatic subarachnoid hemorrhages and demonstrate a rising incidence due to advances in imaging techniques. The study highlights a gender discrepancy in aneurysm occurrence and a higher prevalence in individuals over 30 years old. The research delves into various aspects, including aneurysm localization, diameter, neck dimensions, and rupture status, with a focus on the anterior communicating artery and middle communicating artery as predominant locations. Significant findings include the prevalence of ruptured aneurysms and the impact of arterial hypertension, atherosclerosis, obesity, and diabetes on aneurysm epidemiology. The study also investigates the occurrence of vasospasm, a significant factor in delayed morbidity and mortality in aneurysmal subarachnoid hemorrhage. The utilization of the Glasgow Outcome Scale and other quantification scales aids in understanding the severity and postoperative outcomes of intracranial aneurysms. Challenges such as the incidence of reopenings and postoperative osteomyelitis are addressed, underlining the need for refined protocols and multidisciplinary approaches in treatment. The study’s results contribute to the existing knowledge base on intracranial aneurysms, emphasizing the importance of ongoing research and tailored treatment strategies. The comprehensive nature of this analysis, covering preoperative, intraoperative, and postoperative factors, provides valuable insights into the complex interplay of risk factors and clinical outcomes in patients with intracranial aneurysms.

## Introduction

Intracranial aneurysms (IAs), specifically of the saccular or berry type, constitute acquired lesions and contribute to approximately 80% of nontraumatic subarachnoid hemorrhages (SAH) [1]. IAs affect 5%-10% of the general population [2], with a subset undergoing rupture, leading to severe consequences. Unruptured IAs are infrequently observed in children (0.5%-4.6% of all aneurysms) and appear to manifest more frequently with advancing age [3,4]. The prevalence of individuals harboring an IA in the population aged over 30 years ranges between 3.6% and 6.5% [5-7].

There is an observed gender discrepancy, with women exhibiting a higher likelihood of aneurysm occurrence than men (3:1 ratio of women to men in unruptured cases) [7]. IAs can manifest either as solitary occurrences (70%-75%) or as multiple aneurysms (25%-30%) [8]. The incidence of unruptured IAs appears to be on the rise, possibly attributed to advancements in Magnetic Resonance Angiography (MRA) and Computed Tomography Angiography (CTA) imaging techniques [9]. Regarding SAH resulting from IA rupture, the occurrence is approximately 1.24 times more frequent in women than in men [10], and 2.1 times more common in black individuals compared to whites [11]. Geographical disparities in the annual incidence of SAH are notable, as evidenced by a World Health Organization (WHO) study revealing a tenfold variation in age-adjusted annual incidence in Europe and Asia. This ranged from 2.0 per 100,000 population in China to 22.5 cases per 100,000 in Finland [12]. The risk of IA rupture is contingent upon factors such as size and location, with reported annual rupture rates of 2.7% in the Japanese population [9] and 1.9% in the white population [13].

The precise etiology of IA formation remains elusive; these lesions typically arise due to congenital defects in blood vessel walls, atherosclerotic changes, trauma, or infectious emboli [14]. Crafting an optimal management strategy for IAs necessitates consideration of various factors. Aneurysm-related factors, such as site, size, morphology, the presence of thrombus, and location as depicted in brain imaging, must be evaluated alongside patient-related factors, including age, medical history, previous SAH, and a positive family history of IAs or SAH. Inflammatory and immunological reactions may also be associated with IA formation and rupture [15], albeit not as firmly established as in abdominal aneurysms. Additionally, a decline in both circulating estrogen levels and cerebrovascular estrogen receptor density may contribute to an elevated risk of IA pathogenesis and rupture in women during and after menopause. The impact of these risk factors may result in thickening of the intimal layer, subsequently increasing hemodynamic strain in the more elastic segments of the vessel wall [16]. This, in turn, contributes to the formation and progression of IAs.

A study based on twins estimated the heritability of aneurysmal SAH (ASAH) at approximately 40%, underscoring a significant genetic influence in the development of both unruptured IAs (UIA) and ASAH [17]. This heritability is attributed to a combination of rare, penetrant mutations, and common variants with small effect sizes. Presently, the collective impact of all common variants can elucidate 21% to 29% of the disease, while the complete contribution of rare variants remains undisclosed [18]. Clinical risk factors well-established for both UIA and ASAH include hypertension and smoking [19,20].

## Materials and methods

This article presents an analysis of 346 patients with a cumulative number of 416 IAs treated using microsurgical clipping between 2016 and 2022 at the Department of Neurosurgery, National Institute of Neurology and Neurovascular Diseases in Bucharest, Romania. Each case included in this study has undergone surgical intervention using the clipping method for either a ruptured or UIA. A comprehensive analysis was conducted, focusing on specific variables throughout the preoperative, intraoperative, and postoperative steps of patient medical management, especially surgical characteristics such as surgical approach, aneurysm localization, maximum diameter and neck dimension, clip form, and dimension. Moreover, postoperative complications were discussed and a number of reopenings were acknowledged.

The research aligns with the main principles in the Declaration of Helsinki and obtained approval from the Ethics Committee of the National Institute of Neurology and Neurovascular Diseases in Bucharest, Romania (Ethical Review Board of National Institute of Neurology and Neurovascular Diseases; 560/16.01.2024). Clinical data including risk factors, aneurysm characteristics, and surgical interventions, as well as follow-up details such as postoperative complications and mortality rate, were extracted from relevant files. Processing of all data has been undergone in compliance with current GDPR, and informed consent was obtained from all patients included in this study.

Statistical analysis and figure plotting were conducted using Python version 3.10, developed by the Python Software Foundation, located at 9450 SW Gemini Dr., ECM# 90772, Beaverton, OR 97008, USA. The analysis involved the use of Python libraries such as pandas, numpy, seaborn, and matplotlib.

## Results

**Figure 1:**
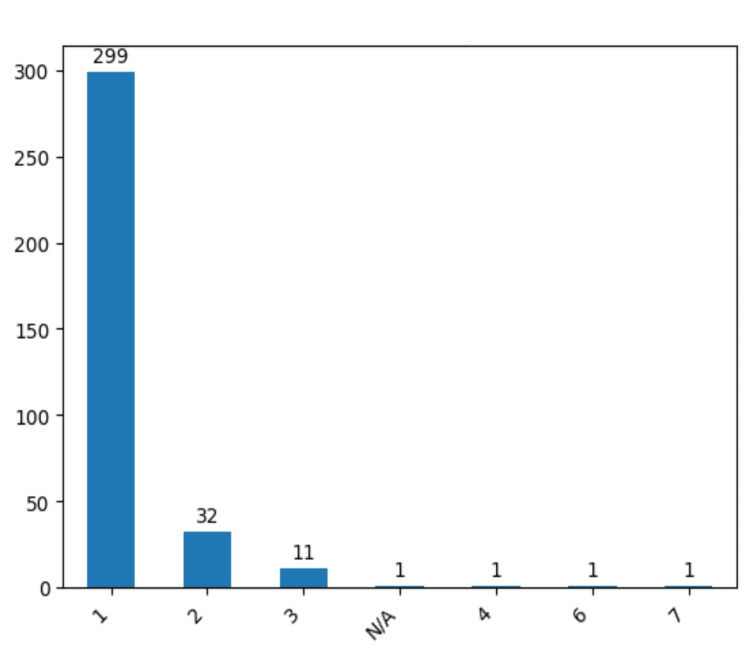
Diagram showing the total number of patients and the number of aneurysms that they presented within the study group. The data have been represented as N = number of values

The study group included a number of 346 patients that presented a cumulative number of 416 IAs. The cohort underwent surgical treatment, focusing on the first aneurysm, primarily focusing on the clipping method. By focusing on the surgical clipping technique as the main type of intervention, our study extended beyond prevalence statistics. It delved into the intricacies of aneurysm management, offering an exploration of the surgical clipping approach's outcomes (Figure 1).

**Figure 2:**
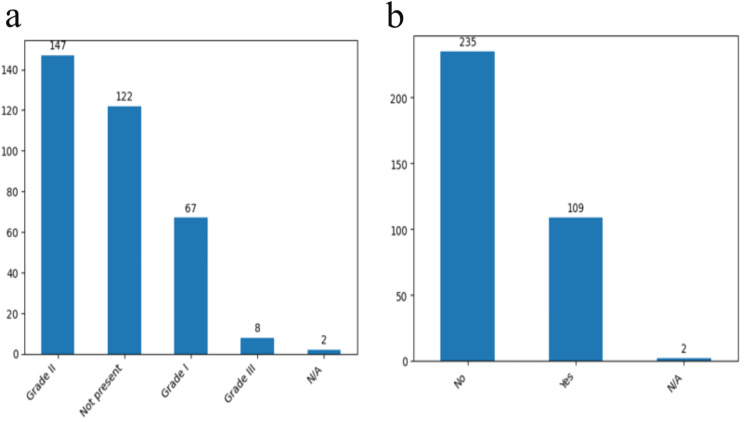
Prevalence of different risk factors Grades of arterial hypertension (a) and the presence or absence of atherosclerosis (b) in the study group. The data have been represented as N = number of values.

In our monocentric study we have confirmed correlations between known IA risk factors and our cohort risk factors. Arterial hypertension, mostly grade II, atherosclerosis and obesity are the most relevant risk factors that we identified, as well as diabetes which cannot be related with the prevalence of IA but rather in influences the intraoperative phase and it is known to increase the risk of postoperative infections (Figures 2a, 2b).

**Figure 3:**
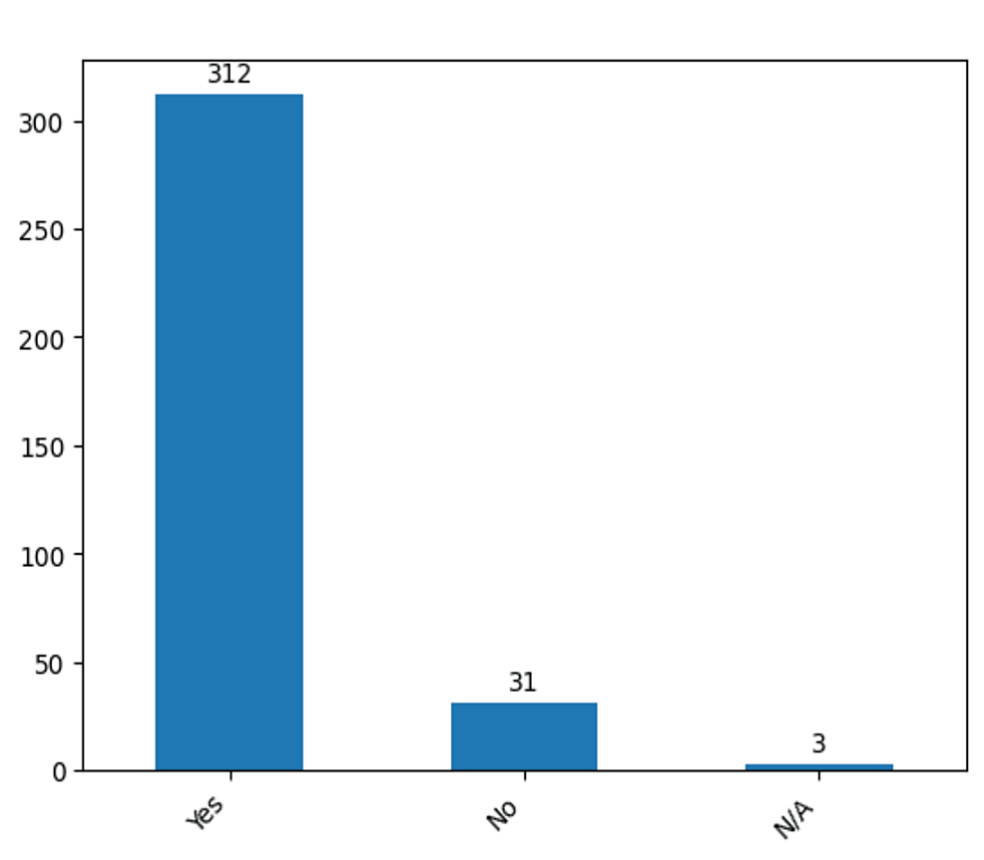
The number of cases that presented preoperative hemorrhage in the study group. The data have been represented as N = number of values

From our dataset that included 346 operated aneurysms, hemorrhage emerged as a predominant characteristic, being observed in 312 cases (90.1%) (Figure 3).

**Figure 4:**
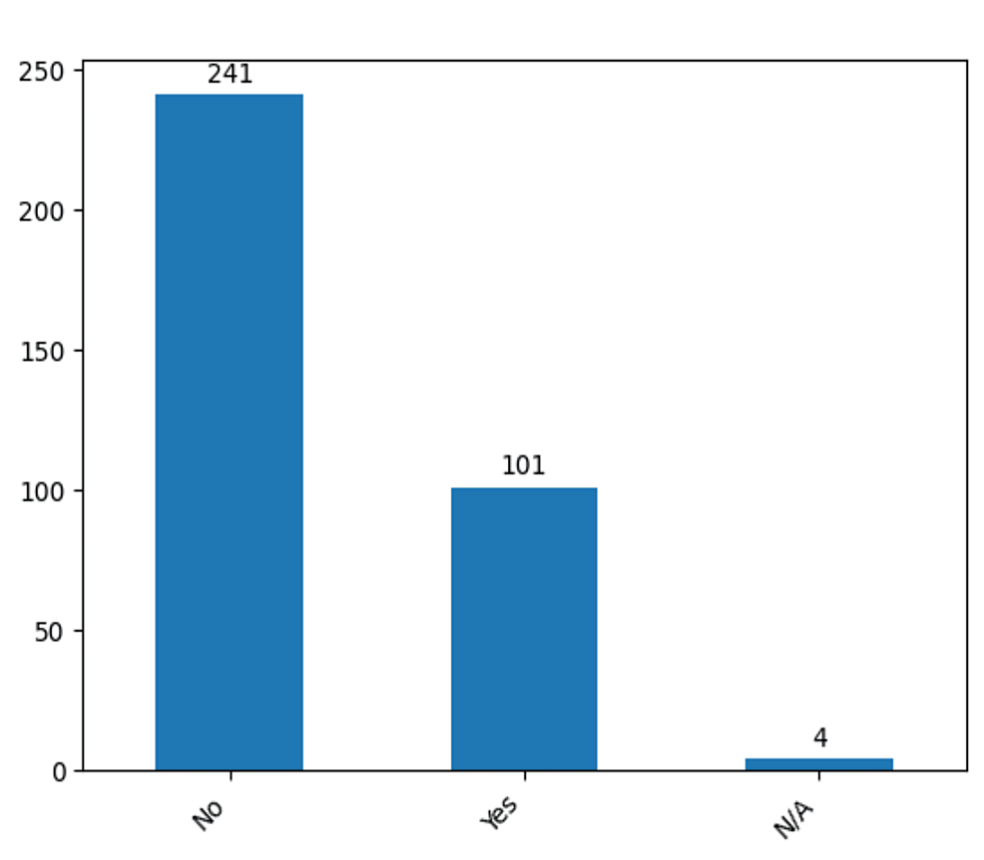
The total number of cases that presented vasospasm preoperative in the study group. The data has been represented as N = number of values.

Interestingly, 101 patients (29.2%) manifested vasospasm, an observable and reversible constriction of cerebral arteries that typically manifests days following SAH as shown (Figure 4).

**Figure 5:**
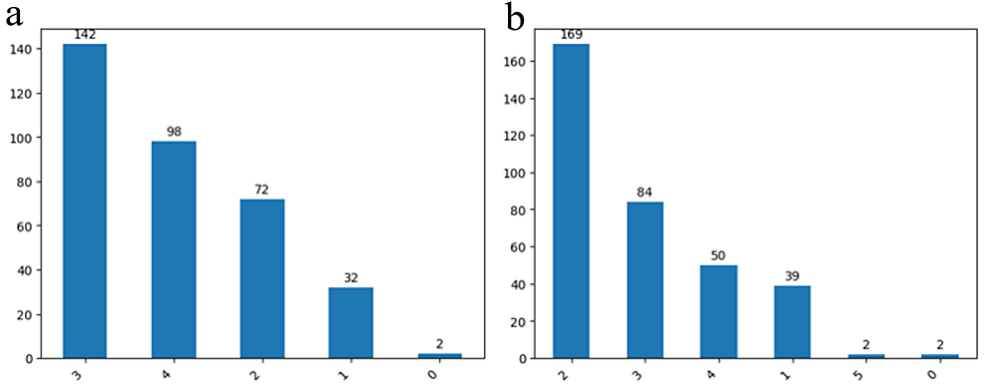
The results of the patients from the study group at admission Fisher score (a) respectively Hunt and Hess score (b). The data has been represented as N = number of values.

The present study employed diverse quantification scales to establish correlations between various variables and the severity of IAs. Our findings indicated that 142 cases (41%) fell within Fisher score 3, 98 cases (28.3%) within Fischer score 4, 72 cases (20.8%) within Fischer score 2, and 32 cases (9.2%) within Fisher score 1, with a mere two cases (0.5%) designated as Fisher score 0. Notably, a substantial majority of cases demonstrated a propensity to be categorized under Fischer score 3 and 4. A similar trend could be observed regarding the Hunt and Hess score, with predominant representation in grades 2 and 3 (Figures 5a, 5b).

**Figure 6:**
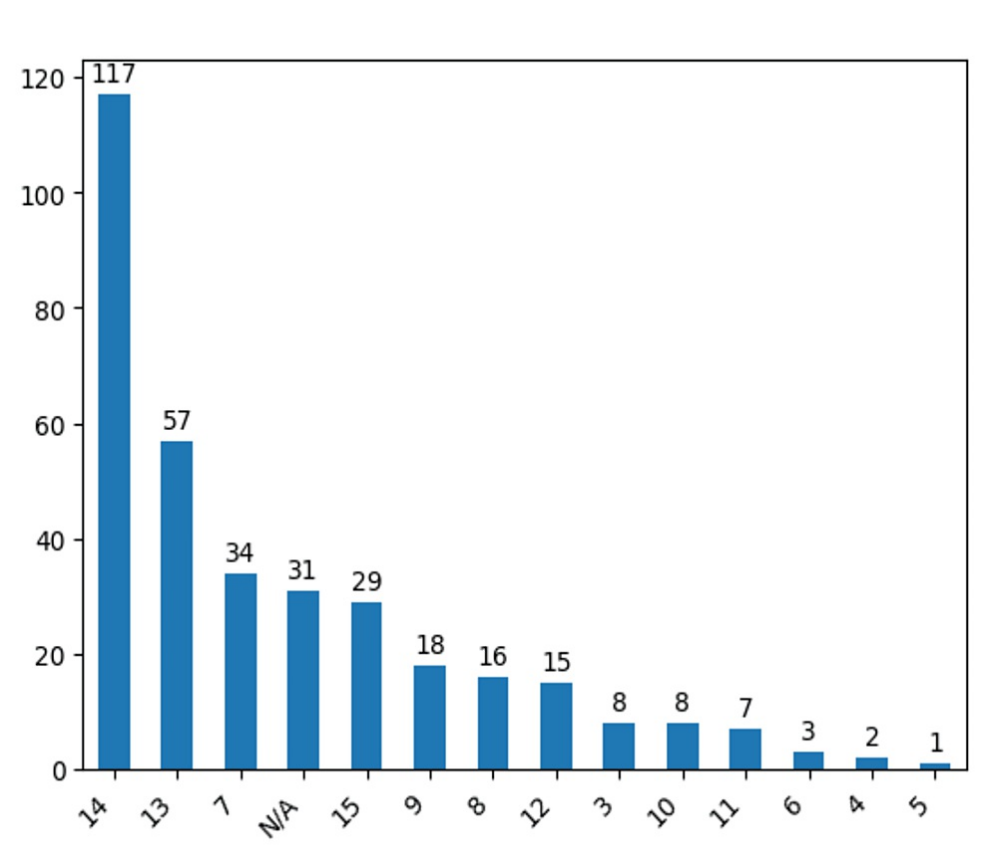
Results using Glasgow Coma Scale at admission for the patients from the study group. The data have been represented as N = number of values.

Additional considerations in our evaluation were aspects such as balance and cognitive disorders, revealing no significant correlation with IA severity. Likewise, preoperative aphasia and hemiparesis exhibited no discernible relevance regarding the apparition or the severity grade of IAs in our study group. We have also Glasgow Coma Scale (GCS) as a clinical outcome prediction scale. Most of the patients had a GCS of 14 at admission, about 117 cases (33.8%) (Figure 6). These findings contribute valuable insights into the varied clinical presentations and associated factors observed in our surgically managed IA cases.

**Figure 7:**
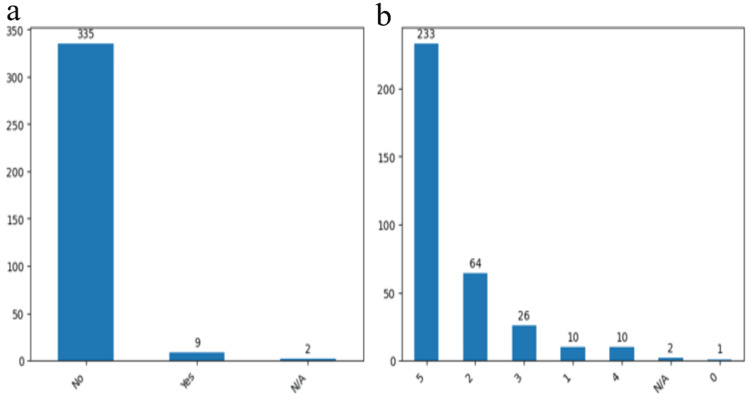
The results obtained after the patients preoperative examination Aphasia presence or absence (a) and hemiparesis assessment using MRC scale (b). The data have been represented as N = number of values.

Furthermore, we conducted an assessment of two additional pertinent parameters both pre- and post-surgery to offer a clinical perspective on the outcomes of the clipping method. Specifically, we examined aphasia and hemiparesis. Preoperatively, the majority of patients, 335 patients (96.8%), exhibited no signs of aphasia, underscoring the prevalent absence of this clinical manifestation. Additionally, a significant proportion of patients, 233 cases (67.3%), fell within the 5th grade of the hemiparesis scale, indicative of a normal functional state prior to the surgical intervention as shown (Figures 7a, 7b).

**Figure 8:**
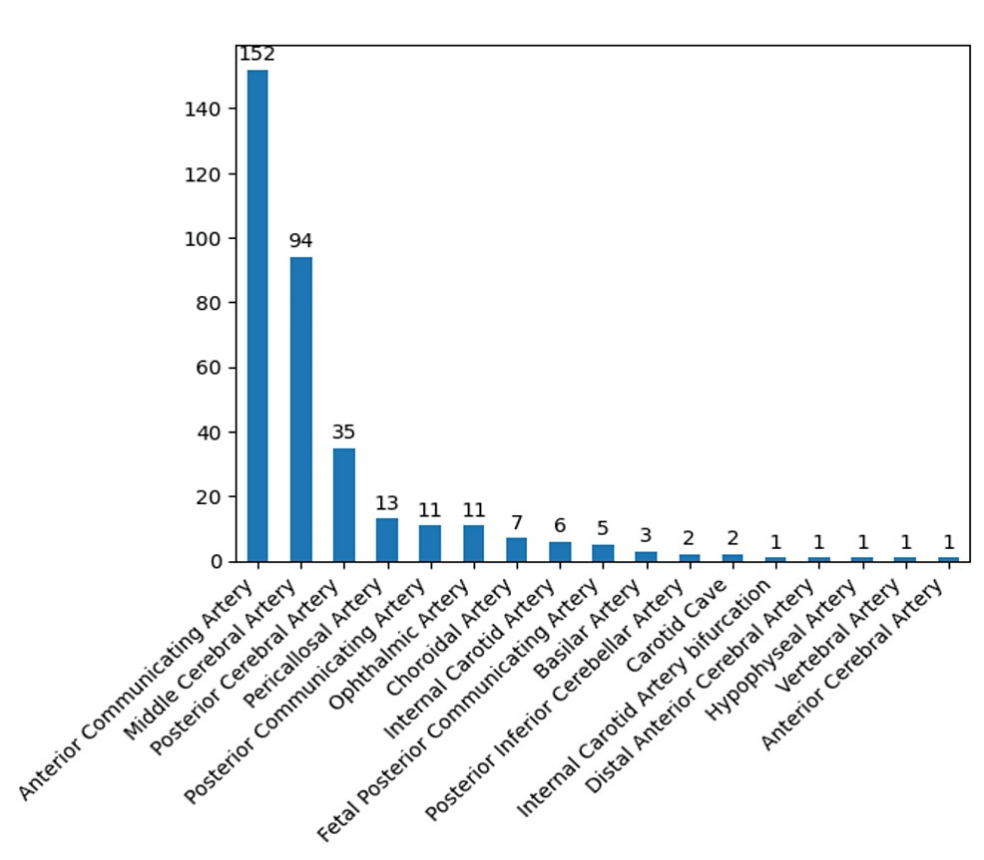
Localization prevalence of the aneurysms in the study group. The data have been represented as N = number of values.

The aneurysm characteristics investigated within our study cohort encompassed localization, diameter, neck parameters, and classification based on rupture status. Predominantly, the anterior communicating artery and middle communicating artery emerged as the two most prevalent localizations, collectively accounting for 246 cases (71%) out of the total 346, indicating a notable prevalence within these regions (Figure 8).

**Figure 9:**
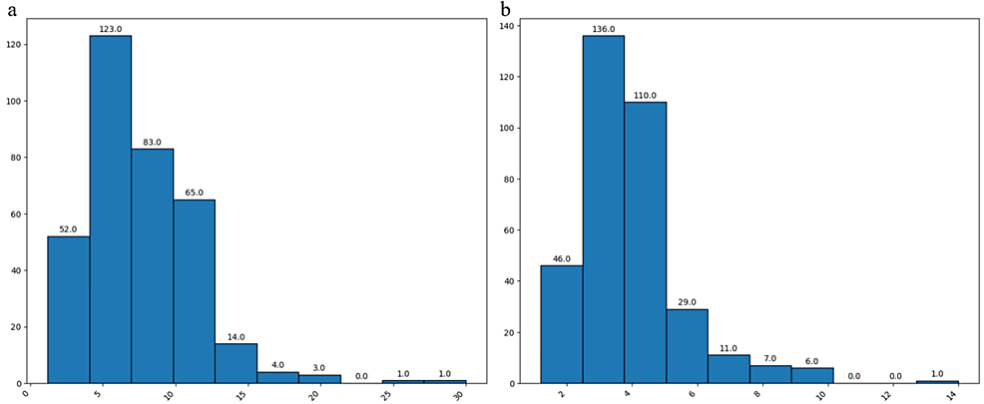
Aneurysm features Results of the surgical treated aneurysms based of their diameter (a) and neck (b) dimension from the patients in the study group. The data have been represented as N = number of values.

Regarding the diameter, our findings indicated a prevalent range of 2-12.5 mm in 323 cases (93.3%), while the neck dimension was primarily situated between 1-5 mm in 292 instances (84.4%). Notably, the majority of cases exhibited diameters within the 5-10 mm interval, with a concurrent concentration of neck diameters in the 3-5 mm range as shown (Figures 9a, 9b).

**Figure 10:**
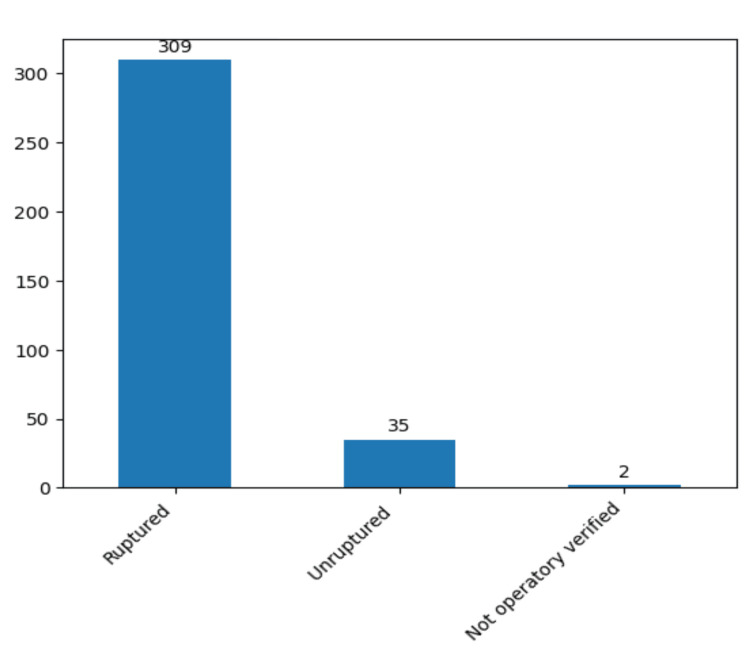
Aneurysms types in our study group. The data have been represented as N = number of values.

The classification of aneurysms based on rupture status revealed a substantial prevalence of ruptured aneurysms within our clinic. Intraoperatively, 309 patients (89.3%) were identified as presenting with ruptured aneurysms as shown by the follow diagram (Figure 10).

**Figure 11:**
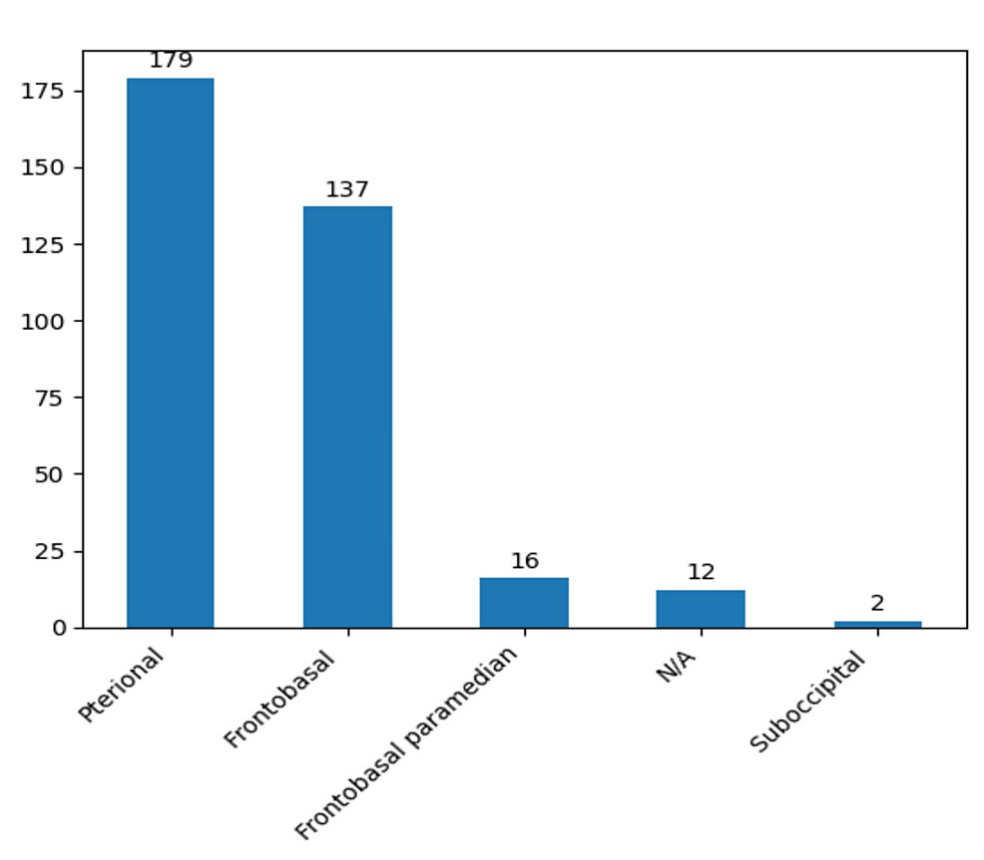
The prevalence of surgical approaches chosen in the study group based on the aneurysm characteristics. The data has been represented as N = number of values.

Our preferred surgical approach, guided by considerations of aneurysm localization, direction, and morphological attributes, primarily involved the utilization of clipping. Craniotomies were predominantly executed through pterional and frontobasal approaches (Figure 11).

**Figure 12:**
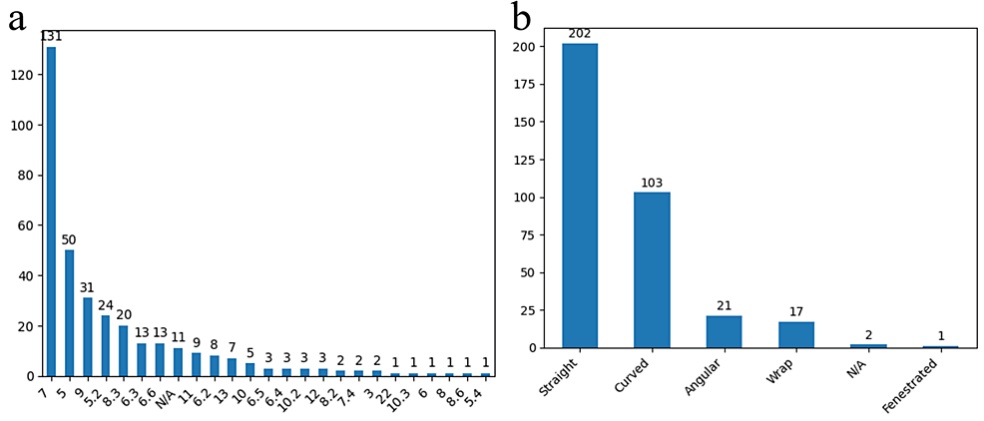
Aneurysmal clip characteristics The clip dimension (a) and form (b) used in the surgically treated aneurysms in the study group. The data have been represented as N = number of values.

The most frequently employed clip dimensions were size 7, evident in 131 cases (37.8%), with a predominant preference for straight-form clips, observed in 202 cases (58.3%) (Figures 12a, 12b).

**Figure 13:**
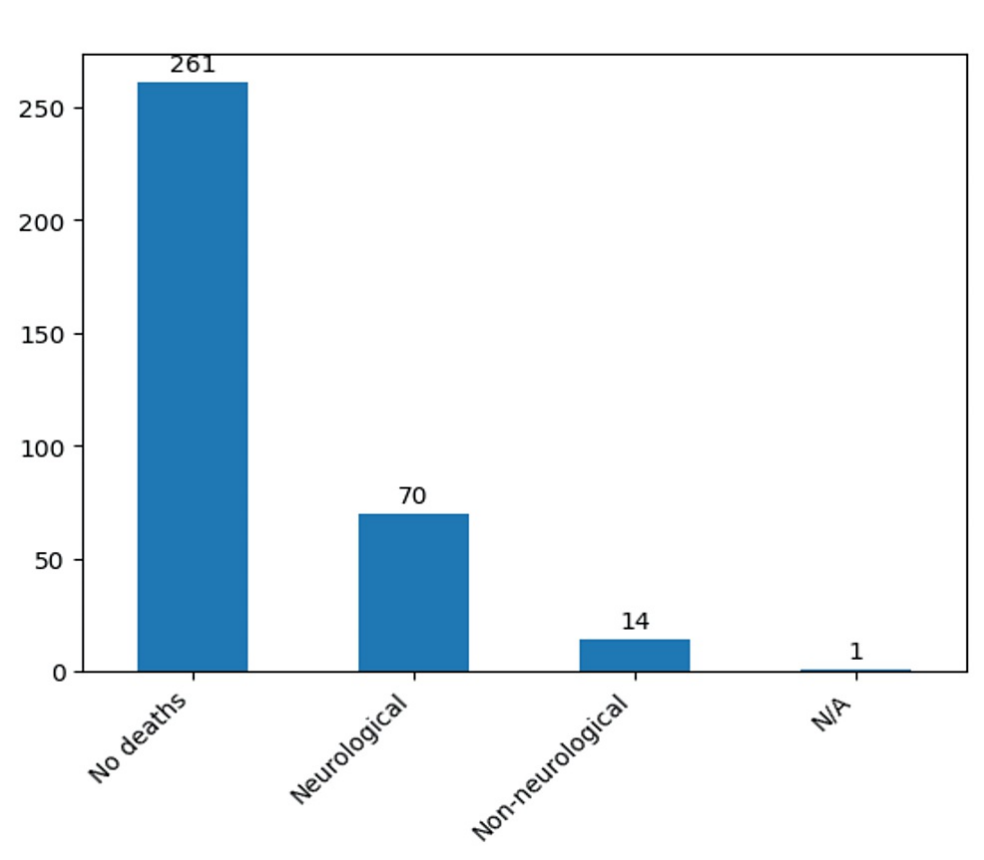
Postoperative causes of death for the patients in the study group. The data have been represented as N = number of values.

Our analysis of postoperative outcomes in the context of surgically treated aneurysms, totaling 346 cases, resulted noteworthy findings. Of this cohort, 261 patients (75.4%) exhibited favorable results, while 84 (24.3%) succumbed postoperatively. Among the latter, 70 individuals (20.2%) experienced neurological causes leading to demise, whereas 14 cases (4%) were attributed to non-neurological factors (with one case unspecified) (Figure 13).

**Figure 14:**
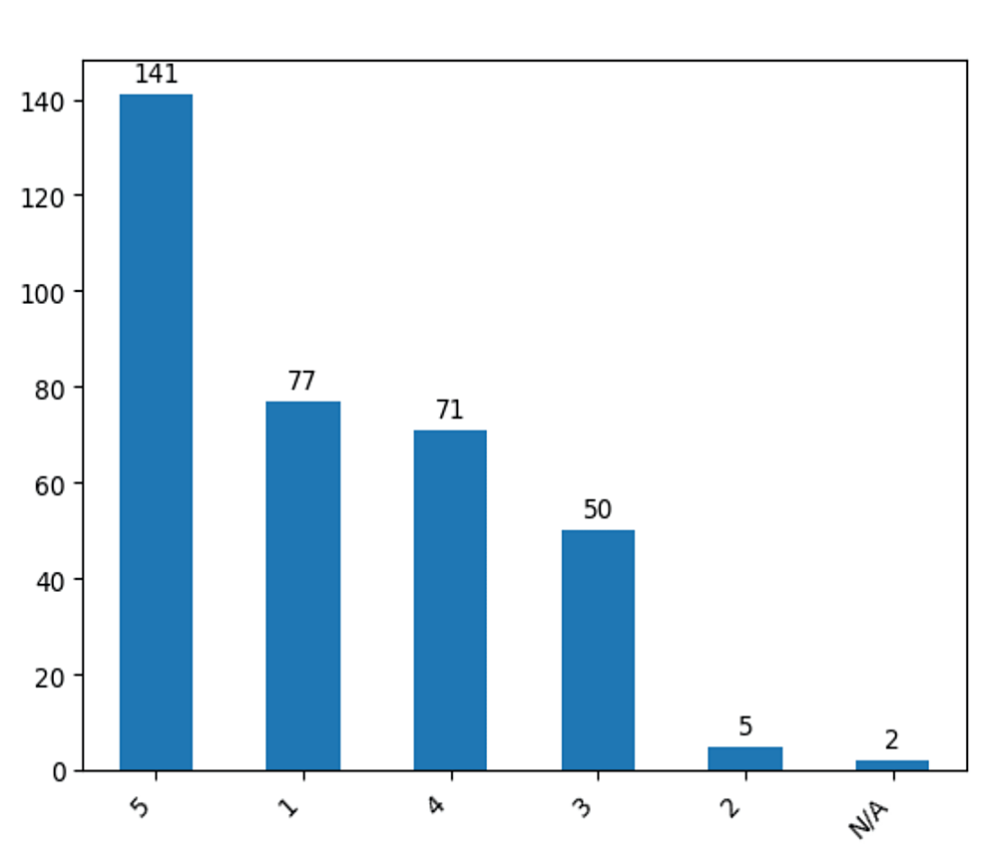
Postoperative results for the patients examined using GOS. The data have been represented as N = number of values. GOS - Glasgow Outcome Scale

The Glasgow Outcome Scale (GOS) was employed for a comprehensive evaluation of postoperative outcomes, with a predominant distribution observed in GOS 5, encompassing 141 patients (40.7%). Subsequently, GOS scores 1 and 4 followed in descending order, with 77 and 71 cases (22.2% and 20.5%), respectively (Figure 14).

**Figure 15:**
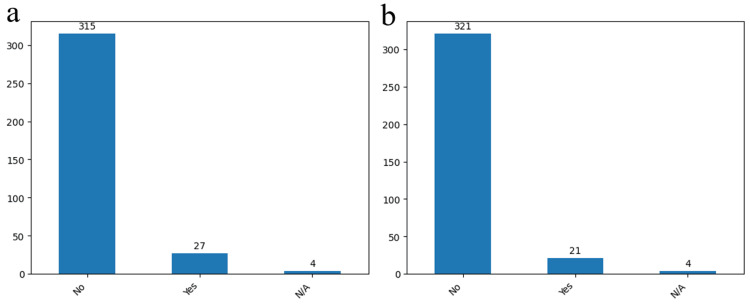
Postoperative clinical results Examination of at postoperative aphasia (a) and visual acuity deficit (b) tests. The data have been represented as N = number of values.

**Figure 16:**
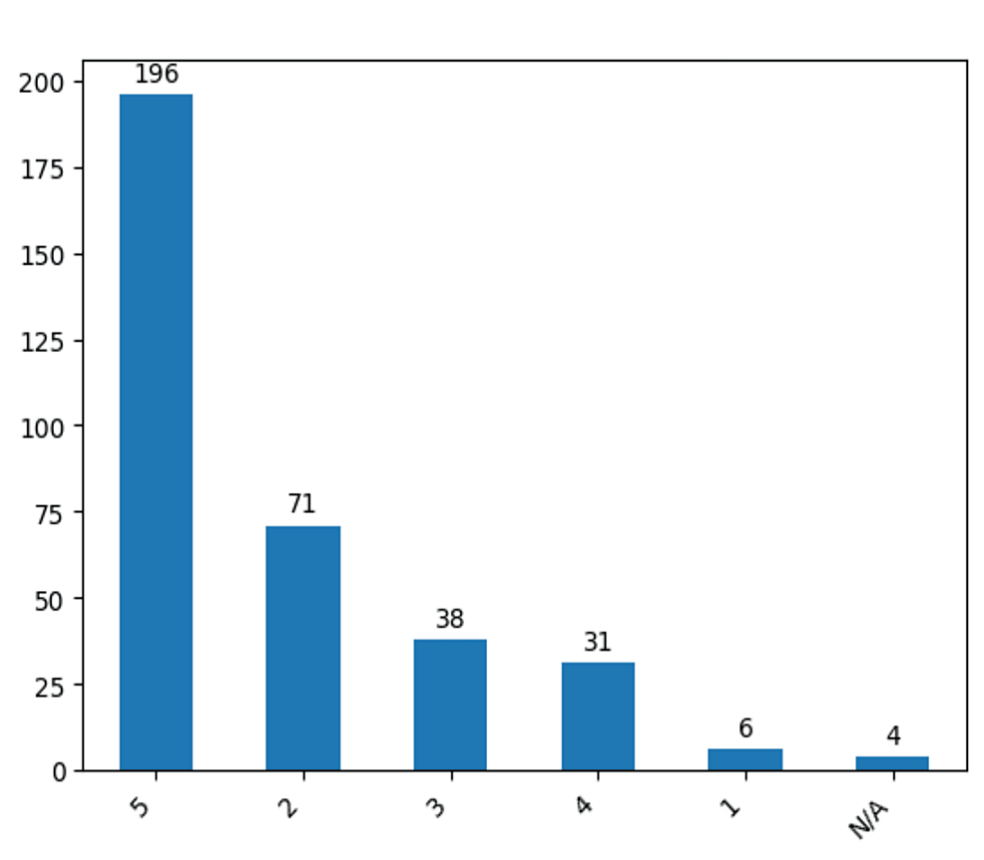
Patient’s results for the postoperative hemiparesis test. The data have been represented as N = number of values.

In our study we have examined relevant parameters such as visual acuity deficit, hemiparesis, and aphasia (Figures 15a, 15b, Figure 16). Remarkably, only 27 patients (7.8%) manifested postoperative aphasia, while a visual acuity deficit was identified in 21 cases (6%). The evaluation of hemiparesis severity, conducted through a dedicated scale, unveiled that the majority of cases exhibited grade 5, indicative of normal muscle strength. Conversely, 71 cases (20.5%) demonstrated grade 2, denoting active movement possible only when gravity was eliminated. Notably, 38 patients (11%) exhibited no resistance in movement, 31 patients (9%) could demonstrate active movement against gravity and resistance, and only six patients (1.7%) revealed a trace of contraction during examination. We can notice a relatively small descending trend considering the preoperative results concluding that the risk of aphasia and hemiparesis postoperative was not accentuated using the clipping technique.

**Figure 17:**
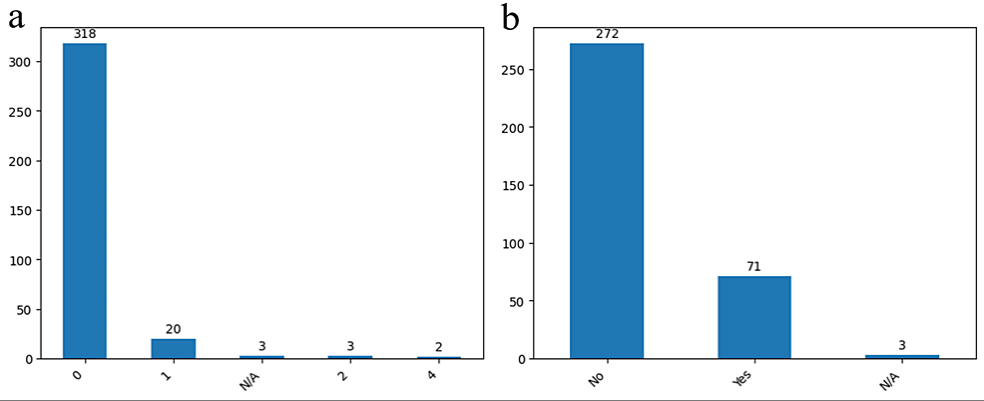
Diagrams showing the number of postoperative reintervations and osteomyelitis Reopenings reported (a) that were needed for further treatment and the number of osteomyelitis cases in the study group (b). The data has been represented as N = number of values.

Furthermore, we explored the incidence of reopenings and cases of postoperative osteomyelitis. Impressively, a mere 25 cases (7.2%) necessitated reopening, attesting to the efficacy of our chosen surgical approach. Conversely, postoperative osteomyelitis manifested in 71 patients (20.5%), prompting further considerations regarding preventative measures and postoperative care protocols (Figures 17a, 17b).

## Discussion

The primary identified clinical risk factors for the formation of IAs encompass female gender, smoking habits, and Autosomal Dominant Polycystic Kidney Disease (ADPKD). Notably, smoking has been demonstrated to significantly elevate both the incidence of new aneurysm development and the growth rate of preexisting ones. The inflammatory impact of smoking on arterial walls weakens the structural integrity, thereby predisposing the vessel to aneurysm formation [21]. The majority of factors influencing aneurysm prevalence appear to correlate with or exert an impact on, either the arterial wall or the hemodynamic loads [22]. A nuanced exploration suggests that the elevated prevalence of cerebral aneurysms and subsequent SAH among females may peak in the post-menopausal period, coinciding with a decline in estrogen levels. The fluctuation in estrogen levels is posited to have consequential effects on vascular integrity, given its supportive role in the normal functioning of the vascular wall [23]. Additionally, anatomical and physiological considerations, such as variations in vessel size and blood flow velocity, may contribute to heightened hemodynamic forces acting on the vessel wall in females, thereby augmenting the risk of prevalence and rupture [24]. Certain hereditary disorders characterized by vascular abnormalities, such as ADPKD or Ehlers-Danlos syndrome (EDS) type IV, exhibit associations with IAs or aneurysmal SAH. Notably, ADPKD has been linked to an estimated aneurysm prevalence approximately five times higher than that observed in the general population. Contrarily, the risks of growth and rupture in ADPKD-related aneurysms have not been observed to surpass those associated with unruptured aneurysms in the broader population [25].

Symptomatic vasospasm constitutes a principal factor in delayed morbidity and mortality among individuals afflicted with aneurysmal SAH, as evidenced by existing research [26]. The severity of such vasospasm is correlated with both the quantity and the duration of exposure to SAH. Notably, approximately 40% to 70% of patients who endure the initial hemorrhagic episode following a cerebral aneurysm rupture experience arterial constriction, with 20%-30% demonstrating neurological deficits [27,28]. Current strategies for preventing vasospasm and subsequent cerebral ischemia post-rupture of cerebral aneurysms are confined to pharmacological interventions and the management of blood pressure, intravascular volume, and blood viscosity. Surgical interventions, including clot removal and irrigation of the basal cisterns, are postulated to diminish the incidence of vasospasm [27,29]. It is widely acknowledged that securing the aneurysm through endovascular coiling or microsurgical clipping is beneficial for the safe management of vasospasm, with the prevalent adoption of 3H therapy, particularly in facilities where angioplasty is unavailable. In our investigation, preoperative clinical grading emerged as a pivotal prognostic element. The sole significant prognostic factor was observed in patients categorized under Fisher grade II, all of whom exhibited a favorable prognosis. This aligns with findings from other scholarly inquiries into prognostic factors following microsurgical clipping of cerebral aneurysms [30].

Advancements in microsurgical and endovascular techniques have significantly enhanced the landscape of management options for brain aneurysms in recent years [31-33]. Despite the progress, the two approaches are acknowledged as inherently divergent, each possessing distinct advantages and limitations, thereby perpetuating a debate regarding the optimal strategy for treating multiple IAs [34]. Notably, a considerable subset of these aneurysms may find successful occlusion through various endovascular techniques instead of traditional microsurgical clipping [35]. However, the definitive determination of the optimal treatment modality remains elusive. Historically, microsurgical clipping has stood as the gold standard for treating both ruptured and non-ruptured IAs. Nevertheless, the expanding realm of endovascular techniques has posed a formidable challenge to this established paradigm. Contemporary perspectives acknowledge endovascular methods as plausible treatment options for selected cases, encompassing single, multiple, and distal-branch IAs [36]. However, certain aneurysms defy effective treatment by a singular technique due to their unique anatomy, location, complexity, and multiplicity. Consequently, the decision-making process for aneurysm treatment necessitates a multidisciplinary approach involving highly experienced personnel, wherein considerations of the aneurysm's characteristics and the patient's individualities guide the determination [37]. Consequently, the treatment of multiple aneurysms may require a hybrid approach, combining both microsurgical and endovascular methods [38]. This underscores the evolving nature of aneurysm management, emphasizing the need for tailored, multidisciplinary strategies based on a nuanced understanding of individual cases.

Our research indicates that the patients who receive thorough clip ligation for a solitary aneurysm at the point of initial admission may not necessitate subsequent imaging for monitoring purposes. Conversely, patients who possess residual or multiple aneurysms warrant extended follow-up surveillance, irrespective of the condition of their clipped aneurysms. Furthermore, there is a noted occurrence of new or de novo aneurysms in patients who have previously experienced an aneurysm at a different site. These particular types of aneurysms might present a heightened risk of SAH [39].

In our investigation, there was no notable increase in the risk of de novo aneurysm formation among patients with multiple aneurysms. This finding contrasts with the results of a follow-up angiographic study by Tsutsumi et al., which re-evaluated the long-term effectiveness of complete clipping of cerebral aneurysms in preventing aneurysm recurrence. In their study, a significant risk of de novo aneurysm formation emerged after a period of nine years, with some instances leading to SAH at a considerably high rate. Given the severe mortality rate associated with SAH, it may be advisable to conduct follow-up examinations approximately nine to 10 years post-surgery, even in patients who have undergone complete aneurysm clipping. This recommendation is based on the potential cumulative risk of aneurysm recurrence, which might surpass 10% [40].

The research conducted by Britz et al. reveals that patients with unruptured aneurysms who do not undergo clipping exhibit a significantly elevated mortality rate compared to those with unruptured aneurysms who receive clipping treatment. Furthermore, patients undergoing clipping for any type of aneurysm demonstrate a progressively increasing annual mortality rate when contrasted with the general population. This trend seems to persist for up to a decade following the clipping procedure, a finding that is corroborated by our own study [41].

Our study main limitation is its retrospective point of view, therefore, data information for each patient was not complete in several cases, especially follow-up details. In plus, our population has been operated using microsurgical clipping alone, no other therapeutic treatment for IAs has been performed, or even combined methods.

## Conclusions

In conclusion, our analysis of 346 IAs subjected to surgical intervention has provided various insights into the intricate interplay of risk factors and prevalent characteristics within this specific patient cohort. The distribution of aneurysms revealed discernible patterns, notably with the anterior communicating artery and middle communicating artery emerging as the predominant localizations. The investigation into aneurysm diameter and neck dimensions unraveled predominant ranges, enriching our comprehension of anatomical variations. Significantly, a noteworthy proportion of cases presented as ruptured aneurysms, underscoring the imperative for timely interventions.

The postoperative evaluation afforded a comprehensive view of outcomes and mortality rates, offering an exhaustive assessment of the efficacy of our surgical interventions. The application of the GOS highlighted favorable results in the majority of cases, accentuating the significance of our treatment approaches. A closer examination of factors influencing clinical outcomes, encompassing visual acuity deficit, hemiparesis, and aphasia, provided pertinent insights into the postoperative neurological landscape.

While our surgical interventions demonstrated overall success, challenges such as the incidence of reopenings and postoperative osteomyelitis demand sustained attention for the refinement of protocols, ultimately enhancing patient care. This study not only contributes to the existing knowledge base on IAs but also underscores the imperative for ongoing research and the adoption of multidisciplinary approaches to optimize treatment strategies.
